# Illustrations of the Effects of Poisons

**Published:** 1834-01-01

**Authors:** 


ILLUSTRATIONS OF THE EFFECTS OF POISONS. By *George Leith Roupell*, M.D. The Plates from original Drawings by An. Melville M'Whinnie, M.R.C.S. Quarto, Part the First. Nicol, Pall Mall, Oct. 1833.

THIS is the first part of a series of drawings, illustrating the effects of the most ordinary irritants on the mucous membranes, and intended to prove useful, not only to the lecturer on forensic medicine and to the general pathologist, but also to those members of the profession, resident in the country, who can meet with few opportunities of witnessing such injuries to the internal surface of the digestive tube, and for which their evidence is often sought in courts of justice. As the plates are taken partly from the effects of poisons on man, and partly from their effects on animals, the author anticipates the following questions. Do poisons exert the same influence on man and on animals ? Do the effects of irritant substances differ so essentially from each other, as to furnish distinctive indications ? The first inquiry, Dr. R. answers by a quotation from Orfila—which, liberally translated, may be thus stated :—" After making more than three thousand experiments on dogs, the only difference between the symptoms and lesions in them and in man, consists in the difference of dose necessary to carry the malady to the same degree—in the influence of the *morale*—and in the relative strength of the two classes of animals—circumstances which operate merely on the intensity of the symptoms—of the organic lesions—and on the duration of the disease produced."

With regard to the second question, viz. whether the effects of different irritants essentially vary ? He can only say that very many substances, and those most frequently employed as poisons, occasion certain changes (when their effects have been fully produced) capable of representation, by which they may be easily distinguished.

" It is not presumed that the appearances met with after death by poison will supersede other modes of investigation, and exclude other kinds of proof, such as the study of symptoms or chemical analysis. My intention in undertaking this work is to afford to the pathologist an additional means of recognizing the consequences of injury of this nature. It is, however, interesting and important to establish the fact, that the effect of certain irritants is peculiar; so peculiar, indeed, that under circumstances favourable to their action, the appearances to which some give rise are evidence sufficient, not only to prove the fact generally that poison had been swallowed, but to satisfy us of the kind of poison actually resorted to.

One object on the score of humanity I have had in view, viz. by the fidelity of the representations to obviate any necessity for the repetition of these experiments; such as presented any unexpected result have been several times performed, and the appearances exhibited have thus been authenticated."—*Preface*, vii.

Mr. Joseph Perry has been engaged to execute the lithography and to colour the engravings—and his part has been ably executed. The drawings, and some of the lithographic impressions, were exhibited at the British Scientific Association, recently held at Cambridge—and the author was fortunate enough to receive, not only the approbation of the medical section, but a grant from the funds of the Association in furtherance of his views.

The illustrative plates, in this Part, are four. The first is designed to shew the effect produced by a large dose of arsenic.

"At two o'clock, p.m. on the seventeenth of January, having kept a large strong dog without food for twenty-four hours, I introduced a drachm of powdered arsenic into his stomach through an opening made in the œsophagus, which was afterwards tied in the manner recommended by Professor Orfila, to prevent the rejection of the poison by vomiting. On removing his muzzle, he displayed such determined ferocity, and was so little affected by the operation, that it was necessary to confine him. In about ten minutes, however, he appeared faint, and in a quarter of an hour he made attempts to vomit.

When visited at four o'clock, two hours after the administration of the poison, he appeared to suffer extremely, frequently varying his position. Retching was urgent and constant, and numerous evacuations from the bowels took place. He continued nearly in the same state until eight minutes before nine, when he died; within seven hours from the time of exhibiting the arsenic.

Examination seventeen hours after death.

The limbs were rigid.

The lungs presented a red appearance, which pervaded their whole tissue, but no change of structure was observed.

The peritoneal surface of the intestines had a rosy hue, the mucous membrane was inflamed in parts, but not intensely.

The stomach and duodenum presented the appearances exhibited and explained in the next page.

Plate I. represents the stomach, part of the œsophagus and duodenum of a dog poisoned by a drachm of arsenic, laid open along their anterior part.

The contracted part towards the centre of the plate denotes the situation of the hour-glass contraction.

The œsophagus is of a rose colour, which results partly from increased vascularity beneath the cuticular lining, and partly from staining by blood effused into the stomach, and brought into contact with the membrane, either by vomiting during life, or accidentally after death.

The peritoneal coat of the stomach and duodenum is red and streaked with numerous blood-vessels, but is free from any deposition upon its surface.

Internally, from the entrance of the œsophagus to the contracted part which marks the commencement of the pyloric extremity (two-thirds of its whole extent) the stomach is of a deep crimson colour, except in some spots which are covered with portions of yellow matter, these are the arsenious acid itself enveloped in mucus, and adhering to the rugæ, but easily detached by the scalpel.

At the contracted part, the deep crimson colour abruptly ceases, and the pyloric end of the stomach and the duodenum only exhibit a few portions of arsenious acid surrounded by a red areola. The diffused yellow tint is owing to the mucous membrane being tinged with bile.

This plate exhibits clearly the simple irritant effect of arsenic. The larger end of the stomach where the hour-glass contraction had forcibly retained the poison in the greatest quantity is intensely and universally inflamed, and the smaller end of the stomach and duodenum, into which lesser portions of the poison had escaped, exhibit only circumscribed patches of inflammation around the spot where the arsenic is deposited.

The mucous membrane has undergone no chemical change by the poison. Neither ulceration nor sloughing has taken place, life was destroyed during the first process of inflammation.

The upper portion of the alimentary canal I have found chiefly inflamed when death has quickly resulted; the lower when life has been prolonged."

The plate itself is beautifully executed and coloured.

The second illustration shews the effect of arsenic on the human stomach. A young woman was brought into Bartholomew's Hospital who had taken arsenic, and who was vomiting and suffering acute pain at the epigastrium. The quantity of arsenic taken was not accurately ascertained. She died in nine hours, notwithstanding the use of the stomach-pump. Were it not that in several places the mucous membrane of the stomach was torn by the stomach-pump, there would be considerable similarity between the two plates.

The third and fourth illustrations are to shew the effects of nitric acid on the stomach of a dog, and on that of a human being.

*Exp.* 1. "At twelve o'clock on the second of March two drachms of concentrated nitric acid were introduced into the stomach of a young dog through an opening in the œsophagus. He exhibited no outward sign of pain at the time, but when placed on the ground he sunk as if exhausted. He made no attempts to vomit. He remained lying on his side in a sort of stupor, when lifted up he fell, but still gave no indication of pain, except that when disturbed he uttered a low moan. At six in the evening he was yet alive, but he was found dead and stiff at ten.

The dissection was made fifteen hours afterwards.

On opening the abdomen a large quantity of coagulated blood of a dark brown colour, was found in the cavity of the peritoneum.

The peritoneum was not inflamed.

The stomach was perforated in two places and disclosed the appearances represented in the following plate.

PLATE III.

Represents the stomach, part of the œsophagus and duodenum of a dog destroyed by nitric acid.

The œsophagus is of a reddish colour.

The stomach is perforated in several places. The edges of the apertures are abrupt and jagged. The mucous membrane at the larger end is in some parts of a bright red; these portions were vascular and but little altered in structure, having been protected apparently by effusion of blood, coagula of which were adherent to them. The coats of the stomach immediately adjoining the perforations were black and thinned, though they retained a considerable degree of firmness.

All the coats of the duodenum and of the pyloric portion of the stomach, except the peritoneal, are of a green colour.

The mucous membrane of the duodenum exhibits its surface full of cracks and fissures. The whole intestine has an appearance of permanent distension, which results from the induration of its coats.

Here it is shewn that acute pain is not a necessary attendant on the most sudden and extensive injury of the stomach, and that perforation may take place and life still continue for many hours.

In this case the œsophagus escaped injury from the acid, which was introduced by a glass tube into the stomach, the redness is attributable to accidental staining.

The colour of the duodenum is owing to the action of the acid on membranes already tinged with bile. I have since this experiment repeatedly produced a similar effect on the dead subject. This is not however the peculiar effect of nitric but is common to all the concentrated mineral acids."

This plate is still more ably executed and beautifully coloured than either of the former.

*Case in the Human Subject.* A lad, 13 years of age, supposing he was going to drink beer, swallowed a mouthful of fluid, which proved to be nitric acid. Acute pain was felt in the mouth and throat. Magnesia was administered, and vomiting was quickly induced. The ejected matters were chiefly food partially digested. Great constitutional depression followed, the chief distress being referrible to the laryngitis. Laryngotomy was performed by Mr. Arnott, but the boy died in thirty-six hours. The lesions, peculiar to this acid, were confined to the tongue, palate, fauces, tonsils, and lining membrane of the pharynx and œsophagus.

"Plate IV. represents the tongue, tonsils, pharynx, and a part of the œsophagus of a boy poisoned by nitric acid.

The basis, edges, and tip of the tongue with the lower part of the œsophagus are seen to be deprived of their cuticular lining. What remains of this lining adherent to the rest of these parts and to the tonsils and pharynx is of a citron colour. The portion which covers the tongue is ragged at its edges, that which covers the pharynx and œsophagus is dry, corrugated, and marked with longitudinal and transverse lines. It was every where capable of being readily stripped from the parts beneath ; these are vascular but not in a high degree.

The edges of the entrance of the glottis are extremely swollen. The epiglottis is shrunk so as to be scarcely recognized.

No traces of the effects of the acid were discovered in the stomach except at the pyloric extremity, where the orifices of the mucous glands were stained of the same citron hue as the cuticular lining of the tongue, pharynx, and œsophagus.

The characteristic colour produced by the action of nitric acid is here well exhibited.

The food contained in the stomach probably defended its coats, but that some of the poison entered it was shown by the stain which was observed near the pylorus, but which was thought too slight to require delineation in this plate.

From noticing the appearances and morbid changes here detected, we may readily believe the account of the extensive portions of membrane stated to have been detached when nitric acid has been taken. In this instance the whole of the membrane acted on by the acid might have been stripped off in one continuous portion. The performance of laryngotomy is an interesting feature in the treatment in this case, but it is no part of my plan to discuss this on the present occasion."

On account of the different modes in which the acid was taken in these two cases, the plates cannot be expected to exhibit the same appearances. As far as the œsophagus is concerned, however, there is sufficient analogy between them.

Of the great merit of the work, in point of fidelity and ability of execution, there can be but one opinion. How far plates, however well executed, can be depended on, in determining the nature of the poison taken, and the certainty of its being taken at all, in a forensic point of view, are questions which we do not feel ourselves capable of answering. The enterprize is assuredly deserving of every encouragement which the profession can bestow.

---

